# Repeatability of gaseous measurements across consecutive days in sheep using portable accumulation chambers

**DOI:** 10.1093/jas/skab288

**Published:** 2021-10-12

**Authors:** Edel O’Connor, Fiona M McGovern, Daire T Byrne, Tommy M Boland, Eoin Dunne, Nóirín McHugh

**Affiliations:** 1 Animal and Grassland Research and Innovation Centre, Teagasc, Athenry, Co. Galway, Ireland; 2 School of Agriculture and Food Science, University College Dublin, Belfield, Dublin 4, Ireland; 3 Animal and Grassland Research and Innovation Centre, Teagasc, Moorepark, Fermoy, Co. Cork, Ireland

**Keywords:** methane emissions, portable accumulation chambers, repeatability, sheep

## Abstract

Portable accumulation chambers (**PAC**s) enable gaseous emissions from small ruminants to be measured over a 50-min period; to date, however, the repeatability of consecutive days of measurement in the PAC has not been investigated. The objectives of this study were 1) to investigate the repeatability of consecutive days of gaseous measurements in the PAC, 2) to determine the number of days required to achieve precise gaseous measurements, and 3) to develop a prediction equation for gaseous emissions in sheep. A total of 48 ewe lambs (c. 10 to 11 mo of age) were randomly divided into four measurement groups each day, for 17 consecutive days. Gaseous measurements were conducted between 0800 and 1200 hours daily. Animals were removed from perennial ryegrass silage for at least 1 h before measurements in the PAC, and animals were assigned randomly to each of the 12 chambers. Methane (CH_4_; ppm) concentration, oxygen (O_2_; %), and carbon dioxide (CO_2_; %) were measured at three time points (0, 25, and 50 min after entry of the first animal into the first chamber). To quantify the effect of animal and day variation on gaseous emissions, between-animal, between-day, and error variances were calculated for each gaseous measurement using a linear mixed model. The number of days required to gain a certain precision (defined as the 95% confidence interval range) for each gaseous measurement was also calculated. For all three gases, the between-day variance (39% to 40%) accounted for a larger proportion of total variance compared with between-animal variance, while the repeatability of 17 consecutive days of measurement was 0.36, 0.31, and 0.23 for CH_4_, CO_2_, and O_2_, respectively. Correlations between consecutive days of measurement were strong for all three gases; the strongest correlation between day 1 and the remaining days for CH_4_, CO_2_, and O_2_ was 0.71 (days 1 and 6), 0.77 (days 1 and 2), and 0.83 (days 1 and 5), respectively. A high level of precision was achieved when gaseous measurements from PAC were taken over three consecutive days. The prediction equation overestimated gaseous production for all three gases: the correlations between actual and predicted gaseous output ranged from 0.67 to 0.71, with the *r*^2^ ranging from 0.45 to 0.71. The results from this study will aid the refinement of the protocol for the measurement of gaseous emissions in sheep using the PAC.

## Introduction

Global temperatures have increased by 1 °C above pre-industrial times and are likely to increase by a further 0.5 °C between 2030 and 2052, if the current rate of increase in global warming is upheld (Allen et al., 2018). During the same timeframe (i.e., up to 2050), the global demand for agricultural products is expected to double; however, the implications of climate change will have large consequences on livestock production and, therefore, the overall food security (Rojas-Downing et al., 2017). Ruminant animals contributed to 39.8% of global agricultural greenhouse gas (**GHG**) emissions through enteric fermentation (FAO, 2019); however, ruminants will still play a vital role in the mitigation of GHG emissions through sustainable livestock production and the adaption of mitigation measures (Rojas-Downing et al., 2017).

To reduce methane (CH_4_) emissions from ruminants, an understanding of the factors associated with these emissions, as well as the ability to measure animals in their natural environment with low-cost, accurate techniques, is required (Robinson et al., 2020). One such technique is using portable accumulation chambers (**PAC**s), a spot sampling method that enables gaseous emissions from sheep to be measured over a 50-min period (Jonker et al., 2018; O′Connor et al., 2021). However, research is required to ensure that these PAC measurements are indicative of actual emissions, are repeatable over time, and can be quantified accurately with as few measurements as possible. Previous studies have investigated the repeatability of gaseous measurements from PAC at a phenotypic (range 0.33 to 0.37; repeated measurements within 3 d; Dominik and Oddy, 2015) and genetic (range 0.33 to 0.39; repeated measurements 14 d apart; Jonker et al., 2018) level. Studies have reported that the repeatability of CH_4_ measurements using both the PAC and respiration chambers declines as the time between measurements increases (Pinares-Patiño et al., 2013; Goopy et al., 2015). The number of days between measurements to achieve a precise measurement is important; this can determine the number of animals that can be measured in that time frame. The current protocol for accurate PAC measurements requires animals’ gaseous emissions to be measured over two measurement periods (Jonker et al., 2018); however, the exact time needed between measurements or the number of days required to achieve a precise measurement has not been investigated. 

The development of a prediction equation for the estimation of gaseous output in ruminants using routinely available data would be beneficial and could save time, labor, equipment, and expense (Patra, 2016); this would allow for the prediction of an animal’s CH_4_ production on a large cohort of animals without the requirement for actual gaseous measurements. Previous prediction models of gaseous output have differed based on the variables used to predict CH_4_ production (Storm et al., 2012), but strong correlations between actual and predicted values have been achieved (Benaouda et al., 2020). To identify and select sheep with lower CH_4_ emissions, a robust methodology is required that can consistently and accurately rank animals divergent on CH_4_ emissions across life stages and season of production. Therefore, the objectives of the current study were 1) to investigate the repeatability of gaseous measurements using the PAC and to ensure that animals consistently rank low or high across consecutive days of measurement, 2) to determine the number of days required to achieve precise measurements, and 3) to develop a prediction equation for gaseous production in sheep.

## Materials and Methods

Data were generated from an experiment undertaken on nulliparous Texel and Suffolk ewe lambs (c. 10 to 11 mo of age) in late winter and early spring 2020 at the Teagasc Animal and Grassland Research and Innovation Centre, Athenry, Co. Galway, Ireland. The study was approved by the Teagasc Animal Ethics Committee (TAEC0496-2020) and the Health protection regulatory authority (AE19132/P098).

### Data collection

The experiment was conducted over 17 consecutive days in 2020. For the purpose of this experiment, CH4, oxygen (O_2_) and carbon dioxide (CO_2_) measurements were obtained using 12 PAC as described by O′ Connor et al. (2021) using a trailer mounted AgResearch designed and constructed Mark III apparatus (www.agresearch.co.nz, Steve Gebbie pers. com.). Briefly, each chamber is an air tight, rectangular shaped aluminium compartment 1.1 m width × 1.07 m average height × 0.77 m length, with an internal volume of 853 L. A sampling valve on top of each chamber allowed for the monitoring of gas measurements while animals were placed in the chambers.

On each of the 17 experimental days, 48 ewe lambs were divided randomly into four measurement groups, each containing 12 animals, and three gaseous measurements were obtained from each animal over a 50-min period between 0800 and 1200 hours daily. Within each measurement group (i.e., 12 randomly selected ewe lambs), each ewe lamb was randomly assigned to 1 of the 12 individual PACs. All ewe lambs were housed indoors for the duration of the experiment and were offered a diet of grass silage ad libitum. One hour before the commencement of measurements, animals were removed from feed (Robinson et al., 2015) and weighed using a Prattley weighing scale (O′ Donovan Engineering Co. LTD, Cork, Ireland).

Upon entry of the lamb into the PAC, measurements for CH_4_ (ppm), O_2_ (%), and CO_2_ (%) were taken at three specific time points: 0, 25, and 50 min after animal entry using the RKI Eagle 2 monitor (Weatherall Equipment and Instruments Ltd, UK). The exact time of each measurement was also recorded. Ambient temperature, atmospheric pressure, and relative humidity were recorded separately for each measurement run. A gas extraction vacuum system is fitted on each of the 12 chambers which allowed for the removal of all residual gases from the chambers between measurement runs. The CH_4_ measurements obtained for each animal over each measurement run were converted to liter/h (l/h) using the equation as described by O′Connor et al. (2021): 


$$\begin{array}{l} CH_{4}\left(l/hour\right)=(\frac{\frac{\left(Methane\;x-Methane\;y\right)}{\left(Time\;x-Time\;y\right)}\times 60)\times \left(853-\left(live-weight\right)\right)}{1,000,000}) \end{array}$$

where CH_4_ (l/hour) is the CH_4_ emissions quantified in liters per hour, Methane_x_ is methane output in ppm at time point x, Methane_y_ is CH_4_ output in ppm at time point y, Time_x_ is the time at time point x, Time_Y_ is the time at time point y, and liveweight is the liveweight of the animal in kilogram.

A similar equation was used to convert the O_2_ and CO_2_ measurements obtained for each animal over each measurement period:


$$\begin{array}{l} Gas\left(l/hour\right)=(\frac{\frac{\left(Gas\;x-Gas\;y\right)}{\left(Time\;x-Time\;y\right)}\times 60)\times \left(853-\left(live-weight\right)\right)}{1oo} \end{array}$$

where gas (l/hour) is O_2_ or CO_2_ quantified in liters per hour, Gas_x_ is the percentage O_2_ or CO_2_ at time point x, Gas_y_ is the percentage O_2_ or CO_2_ at time point y, Time_x_ is the time at time point x, Time_y_ is the time at time point y, and liveweight is the liveweight of the animal in kilogram.

For each of the above equations, the final gas volume obtained in l/hour can be extrapolated up to a g/d value as described by O′Connor et al. (2021):


$$\begin{array}{ll} CH_{4}\left(g/day\right)= & \;CH_{4}\;(l/hour)\times (Press\;\times 0.1)\\ & /(8.3145\times \left(Temp+273.15\right))\times 16\times 1440 \end{array}$$

where CH_4_ (l/hour) is CH_4_ emissions quantified in liters per hour, press is the pressure expressed in hectopascals, temp is the temperature expressed in degrees Celsius, 16 is the molecular weight of CH_4_, and 1440 is the number of minutes in the day. This equation was also used for the calculation of CO_2_ and O_2_; however, the molecular weight was changed from 16 in the case of methane to 44 for CO_2_ and 32 for O_2_.

### Chemical analysis

Representative samples of the silage offered were collected daily. Samples were dried at 60 °C for 48 h using a Memmert “Excellent” forced air circulation oven (Memmert GMBH., Schwabach, Germany) to determine dry matter (**DM**) content, which on average was 22.7 ± 1.3% DM. Samples were bulked by day of measurement and were subsequently analyzed for DM, ash, neutral detergent fiber (**NDF**; Van Soest, 1963), acid detergent fiber (**ADF**; Van Soest, 1963), and crude protein (**CP**; Leco FP‐428; Leco Australia Pty Ltd., Baulkham Hills, New South Wales, Australia).

### Statistical analyses

To quantify the effect of animal and day variation on gaseous emissions, between-animal, between-day, and error variances were calculated for each gaseous measurement (i.e., CH_4_, O_2_, and CO_2_) using a linear mixed model in ASREML (Gilmour et al., 2009), with both animal and day included as random effects. A log likelihood ratio test was undertaken on nested models to determine whether the inclusion of both animal and day as random effects improved the fit of the data. The proportion of total variance explained by animal (H_ani_) was calculated as:


$$\begin{array}{l} H_{ani}=\frac{\sigma_{ani}^{2}}{\sigma_{ani}^{2}+\sigma_{day}^{2}+\sigma_{e}^{2}} \end{array}$$

where $\sigma_{ani}^{2}$ is the between-animal variance, $\sigma_{day}^{2}$ is the between-day variance, and $\sigma_{e}^{2}$ is the error variance. The between-animal coefficient of variation (CV) was calculated as:


$$\begin{array}{l} CV=\frac{\sigma_{ani}}{\mu } \end{array}$$

where µ is the mean of the gaseous measurement and $\sigma_{ani}$ is the between-animal standard deviation of the gaseous measurement under investigation.

The number of days required to gain a certain precision (P_*n*_) with a 95% confidence interval (CI) for each gaseous measurement was calculated as:


$$\begin{array}{l} Pn=1.96\;x\sqrt{\frac{\sigma_{e}^{2}}{n_{\in_{\left(1,17\right)}}}} \end{array}$$

where *n* is the number of days that varied from 1 to 17 and $\sigma_{e}^{2}$ is the error variance. The repeatability of gaseous measurements was calculated using PROC VARCOMP with liveweight and humidity as fixed effects and animal and date of measurements included as random effects. The correlation between each measurement day and all other days was calculated using PROC CORR (SAS Inst. Inc., Cary, NC). Similarly, the regression coefficients (*R*^2^) between each measurement day were calculated using PROC REG (SAS Inst. Inc., Cary, NC).

The ability of the model to predict future gaseous emissions was undertaken, whereby the dataset was stratified by animal and date of measurement and randomly divided into four evenly sized groups. Cross-validation was performed on each of the four groups separately. A prediction equation was developed using a linear mixed model in PROC MIXED (SAS Inst. Inc., Cary, NC), whereby CH_4_ (expressed as liters per hour) was the dependent variable; date of measurement, measurement group, breed, age, and liveweight of the animal, relative humidity, temperature, and pressure were included as fixed effects; and animal was included as a random effect. Gaseous measurements from one stratum at a time were masked with the excluded records predicted on the entire remaining dataset. The goodness of fit of the model on the excluded data was determined by the square of the correlation between the predicted and actual gaseous measurements (*r*^*2*^), as well as the root mean square error (**RMSE**), and was averaged across all four cross-validation datasets.

## Results

The average liveweight of the ewe lambs across the 17 experimental days was 46 ± 4.0 kg. The average temperature, pressure, and humidity across the 17 d period was 8.33 ± 2.45 °C, 1,000.05 ± 15.79 hPa, and 76.68 ± 8.66%, respectively. The mean output of CH_4_ measured across the 50 min was 0.0094 ± 0.0028 l/h and ranged from 0.0018 to 0.020 l/h across the 17 consecutive days. This value could be extrapolated to a grams per day value of 9.19 ± 2.74 g/d. The average O_2_ consumed over the 50-min period was 0.36 ± 0.13 l/h, whereas the mean CO_2_ produced was 0.22 ± 0.08 l/h, which could be extrapolated to a value of 591.40 ± 206.90 g/d CO_2_. Silage quality analyses results (mean ± SD) were as follows: NDF: 517.4 ± 40.9 g/kg DM, ADF: 317.1 ± 26.3 g/kg DM, CP: 121.1 ± 5.6 kg DM, and Ash: 88.2 ± 13.9 g/kg DM.

### Inter-day variation

**Figure 1.**
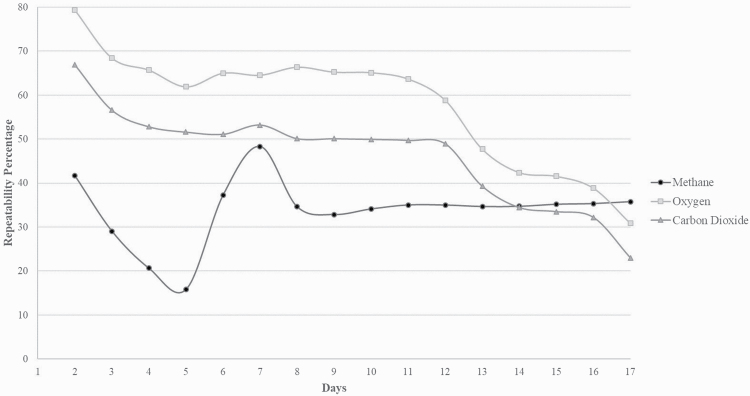
The repeatability of consecutive days of gaseous measurements for methane and carbon dioxide production and oxygen consumption.

For CH_4_, the proportion of total variation, calculated across all 17 d, attributed to the between-animal and between-day variance was 23% and 39%, respectively. Similarly for both CO_2_ and O_2_ emissions, a greater proportion of the total variation was associated with the between-day variance compared with the between-animal variance (Table 1). The CV for between-animal variance was 0.15 for CH_4_, 0.22 for O_2_, and 0.17 for CO_2_. The repeatability of gaseous emissions across the total 17 d was 0.36, 0.31, and 0.23 for CH_4_, CO_2_, and O_2_, respectively. The repeatability of CH_4_ emissions across the individual 17 d ranged from 0.16 (five consecutive days of measurement) to 0.48 (seven consecutive days of measurements; Figure 1).

**Table 1.** Mean gaseous output (liters per hour), between-animal variance, between-day variance and error variance (SE in parentheses), coefficient of variation (CV), and the proportion of total variation explained by between-animal ($H_{ani}$) for each of the three gaseous measurements

Variable	Mean, l/h	Animal variance, l/h	Day variance, l/h	Error variance, l/h	CV	$H_{ani}$%
Methane	0.0094	0.0000018 (0.0000040)	0.0000030 (0.0000011)	0.0000077 (0.0000015)	0.1467	23.08
Oxygen	0.3582	0.0056 (0.0012)	0.0069 (0.0025)	0.0051 (0.0026)	0.2154	31.80
Carbon dioxide	0.2194	0.0014 (0.0003)	0.0023 (0.0008)	0.0021 (0.0001)	0.1746	23.70

**Figure 2.**
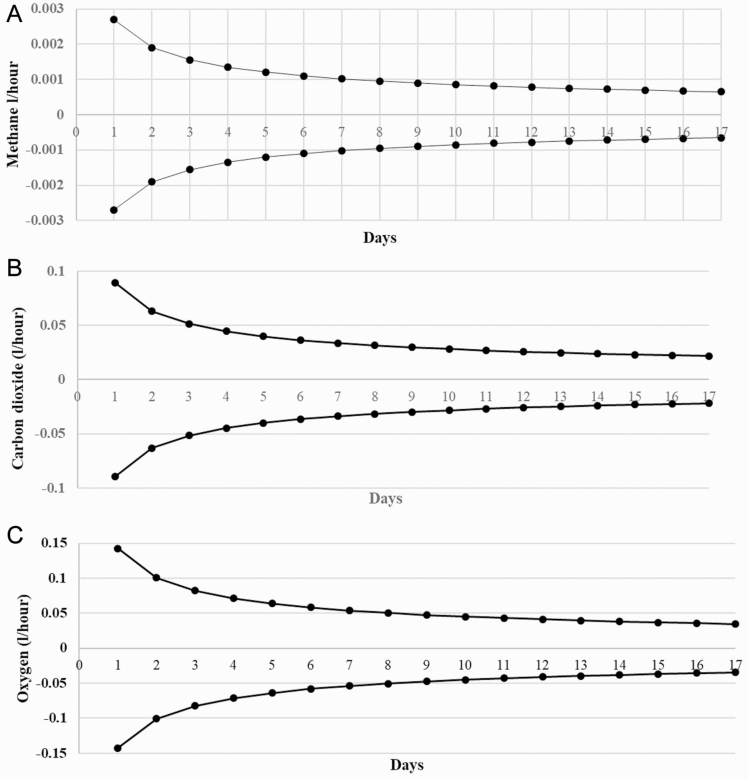
The number of days required to gain a certain precision (P_n_) with a 95% confidence interval of (a) methane, (b) carbon dioxide production (l/h), and (c) oxygen consumption

The number of consecutive days measurements required to achieve a defined level of precision, defined as the 95% CI range, within which the gaseous emissions differed from the average of the 17 d period is shown in Figure 2. When the average CH_4_ emissions were extracted from seven consecutive days, the average of CH_4_ emissions was expected to be within ± 0.0010 l/h of the average of the 17 d of CH_4_ emissions (Figure 2a); when the number of days was reduced to two consecutive days, an average precision of ± 0.0019 l/h was achieved. For O_2_ consumption and CO_2_ gaseous emissions, the average precision achieved from seven consecutive days of measurements was ± 0.05 and ± 0.03 l/h, respectively (Figure 2b and c).

### Intra-day correlations

**Figure 3.**
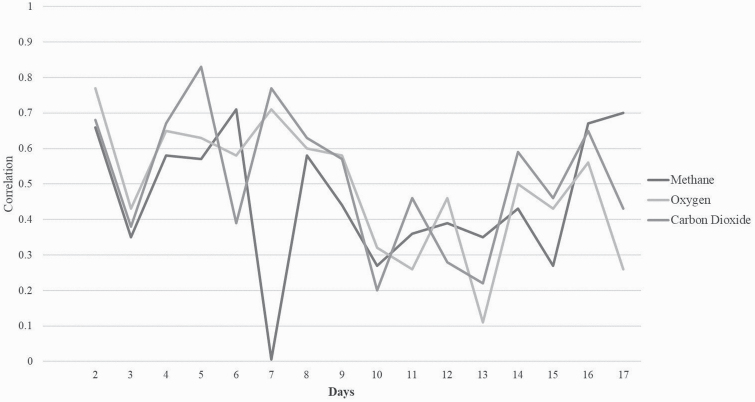
The correlations between day 1 and the remaining days for methane and carbon dioxide production and oxygen consumption.

The correlation between day 1 and all other days for CH_4_ measurements ranged from 0.005 (days 1 and 7) to 0.71 (days 1 and 6; Figure 3). The corresponding regression coefficients between day 1 and all other days for CH_4_ ranged from 0.26 (SE 0.18; days 1 and 8) to 1.21 (SE 0.27; days 1 and 5). With the exception of measurements taken on day 7, a trend was observed, whereby the correlations between day 1 and all other days tended to weaken as the time between the measurements increased; however, the correlations strengthened again on the final 2 d of measurement (days 16 and 17). The strongest correlation between all the other days was observed between days 3 and 4 with a correlation of 0.81, whereas the weakest correlation (0.01) was calculated between days 6 and 11. Correlations between consecutive days remained strong with the exception of the correlation between days 7 and 8 (−0.11).

The correlations between O_2_ measurements across 17 d are shown in Figure 3. The weakest correlation between day 1 and all other days was 0.11 (days 1 and 13), with the strongest correlation of 0.77 (days 1 and 2). The corresponding regression coefficients between day 1 and all other days ranged from 0.56 (SE 0.27; days 1 and 13) to 1.42 (SE 0.27; days 1 and 6). Correlations remained strong for the first 9 d before weakening and increasing again on day 14. The correlations between all days ranged from 0.14 (days 2 and 13) to 0.93 (days 7 and 8). On consecutive days of measurement, correlations remained strong with the exception of the correlation between days 2 and 3 (0.18).

The correlation between day 1 and all other days of CO_2_ measurements ranged from 0.20 (days 1 and 10) to 0.83 (days 1 and 5; Figure 3). The corresponding regression coefficients between day 1 and all other days ranged from 0.85 (SE 0.31; days 1 and 3) to 2.46 (SE 0.65; days 1 and 5). A trend was observed with the correlation between day 1 and the remaining days remained strong until day 9 before weakening; however, the correlations strengthened again from day 14 onwards. The correlation between all other days ranged from −0.05 (days 2 and 3) to 0.91 (days 15 and 19). With the exception of the correlation between days 2 and 3, correlations between consecutive days of measurements remained strong.

### Prediction equation

For CH_4_ production, the mean predicted value calculated using the prediction equation was 0.036 l/h, and the correlation between the actual and predicted values was 0.71, with a corresponding *r*^2^ of 0.51 and an RMSE of 0.001 l/h. The predicted mean value for O_2_ consumption was 1.50 l/h, which differed considerably from the actual O_2_ consumption mean of 0.35 l/h. The correlation between the actual and predicted O_2_ consumption was 0.67, with an *r*^2^ of 0.45 and an RMSE of 0.06 l/h. For CO_2_ production, a strong correlation (0.67) was estimated between the actual and predicted values; the corresponding *r*^2^ was 0.45 and the RMSE was 0.04 l/h. Across all three gaseous emissions, the predicted equations tended to overestimate the gaseous emissions ranging from 0.027 to 1.15 l/h for CH_4_ and CO_2,_ respectively.

## Discussion

To ensure the consistent and accurate ranking of CH_4_ divergent animals across season of production and life stages, a robust methodology is required that will enable the identification and breeding of sheep with lower CH_4_ emissions. Therefore, the objectives of the present study were to investigate the repeatability of gaseous measurements using PAC and to ensure that animals rank consistently high or low across consecutive days of measurement, thereby allowing low CH_4_-emitting sheep to be identified.

### Repeatability of PAC measurements

Investigating the repeatability of PAC measurements is key to determining when a second PAC measurement is needed and to ensure increased accuracy of PAC measurements. A high repeatability calculated on the PAC measurements across the 17 d of measurement would indicate that a second measurement would not be required within this timeframe. The repeatability of the three gaseous emissions estimated in the present study (0.23 to 0.36) was similar to those reported for the percentage feed eaten by adult ewes (0.34; Robinson et al., 2014b) but lower than the repeatability calculated for liveweight in sheep (0.93 and 0.80, respectively; Pinares-Patiño et al., 2013). Feed intake is the main driver of CH_4_ production, accounting for 78% of variation (Benaouda et al., 2020), and, therefore, one would expect repeatability of gaseous emissions and feed intake to be similar; however, this was not the case in this study. The repeatability of the three gaseous measurements from the present study aligns with those previously reported at both phenotypic (repeat measurements within 3 d; Dominik and Oddy, 2015) and genetic (repeat measurements within 2 to 14 d; Robinson et al., 2010; Jonker et al., 2018) levels using the PAC chambers and indicates that a second PAC measurement is required to increase accuracy. In contrast, the repeatability measurements calculated using the PAC were considerably lower than those calculated using the Greenfeed system (0.78 to 0.79; Arbre et al., 2016; Manafiazar et al., 2016) or respiration chambers (Pinares-Patiño et al., 2013; Pickering et al., 2015). Lower repeatability estimates would be expected for the PAC as it is a point in time measurement compared with the Greenfeed system and respiration chamber, which can take multiple measurements across a 24-h period and, therefore, able to detect fluctuations in CH_4_ production throughout the day. Lower repeatability estimates have been found for gaseous measurements taken on the same or consecutive days due to the animals having less opportunity to eat and due to the variation in time and amount of feed eaten (Pickering et al., 2015; Robinson et al., 2020).

Greater between-day variances, as opposed to between-animal variances, were calculated for all three gases in the present study which corroborates to a previous study on CH_4_ emissions measured using the SF_6_ tracer technique in dairy and beef cattle that had ab libitum access to feed (Boadi and Wittenberg, 2002; Garnsworthy et al., 2012). In contrast, Blaxter and Clapperton (1965) showed similar values for the between-day and between-animal variance using respiration chambers. This is in agreement with the phenotypic correlation between two consecutive days of CH_4_ grams per day measurements in the respiration chamber was 0.95 (Donoghue et al., 2016), thus suggesting that repeated measurements on the same animals over a number of days would be required using PAC measurements to achieve the same level of precision as achieved by the respiration chambers (Grainger et al., 2007).

### Precision

Increasing the number of repeated measurements of a trait increases the precision of the measurement (Bergman, 2018), which was similar in the present study, whereby precision increased daily from day 2 to 17. However, the practicality of recording this number of measurements, especially on experiments containing large numbers of animals must also be considered; for example, if measurements were required for 7 consecutive days, then only 60 animals gaseous emissions would be measured in a week compared with 420 animals if gaseous emissions were taken 1 wk apart. Using the selection index theory (Hazel, 1943) and assuming a heritability of 0.29 for methane emissions (Pinares-Patiño et al., 2013), a greater level of accuracy and a greater response to selection for CH_4_ emissions would be achieved by measuring seven closely related animals than by estimating the gaseous emissions on the same animals over seven consecutive days.

An increase in precision is associated with increasing the number of days of measurement in the current study, which is unsurprising assuming that the variance of the data does not change, as the number of measurements that increase the SE is expected to reduce proportionally (Byrne et al., 2017). Given that the difference between the lowest and highest CH_4_-emitting sheep ranges from 15% (using PAC; Robinson et al., 2015) to 34% (using respiration chambers; Pinares-Patiño et al., 2011), this would result in a difference ranging from 0.0014 to 0.0031 l/h in CH_4_ emissions between greater and lesser emitters in the current study. Based on the precision calculated in the present study, three consecutive days of PAC measurements allow for a 17.5% difference between extremes to be detected; this means that difference between the extreme animals can be detected based on the difference found in Robinson et al., (2015) study. However, when animals are measured for seven consecutive days, the difference between extremes reduces to 11.2%, which is less than the differences detected between extreme animals divergent for CH_4_ by Robinson et al. (2015) which means that the difference between extremes cannot be detected.

### Inter-day correlations

Strong to moderate correlations were estimated between day 1 and the following 5 d of CH_4_ measurements, indicating that the animals ranking remained relatively consistent throughout this period. This aligns with the results from the repeatability analysis in the current study, which showed the greatest repeatability for 7 consecutive days of measurement (0.48), highlighting that there is no need for repeated measures within this time frame. From day 7 on, the correlations declined steadily and indicated that the ranking of the animals changed. This may be partly explained by differences in the environmental conditions; for example, the weakest correlations were recorded between days 1 and 7 (0.005), which coincided with the lowest temperature and relative humidity but greater pressure relative to day 1. Lockyer and Champion (2001) showed that changes in temperature and humidity could alter the amount of CH_4_ produced by sheep. With the exception of day 7, the correlation between day 1 and the remaining days tended to weaken as days between measurements increased; however, an increase in the correlation was observed on the final 2 d of measurement. For O_2_ and CO_2_ measurements, however, the correlations between day 1 and all other days remained moderate for the first 9 d before declining.

There is a paucity of studies that have estimated gaseous measurements from PAC across consecutive days over an extended period of time; rather, previous studies have tended to focus on the correlation been gaseous measurements over a larger time frame (Goopy et al., 2015; Robinson et al., 2016; Jonker et al., 2018) and have reported moderate to strong correlations at both genetic (0.44 to 0.55) and phenotypic levels (0.64) when the measurements ranged from 14 d to 1 yr apart. However, results from the current study suggest that a second gaseous measurement should be taken at least a week apart from the first measurement to avoid any residual correlation between the amount of feed eaten before the test and the CH_4_ measurement (Robinson et al., 2014a) and to minimize the disruption to the grazing pattern of the animals (Robinson et al., 2020).

The present study investigated the consistency of the ranking of animals based on high and low CH_4_-emitting lambs over 17 consecutive days of measurement in the PAC. Three of the lowest CH_4_-producing lambs ranked in the 10 lowest emitters across 75% (13 d) of the time, while one lamb ranked as the lowest emitter 41% of the time (7 d). Similar to the lowest CH_4_ emitters, three animals ranked in the 10 greatest emitters 76% of the time. This highlights the consistency of the ranking of the animals in the PAC across time.

### Predicting CH_4_

Prediction equations have been successfully developed for predicting CH_4_ emissions across various species including sheep (Goopy et al., 2015), beef (Yan et al., 2000), and dairy (Yan et al., 2006) animals and allow for an accurate prediction of methane emissions without requiring routinely CH_4_ measurements, thus saving time and expensive equipment (Patra, 2016). A similar CH_4_ prediction model as developed in the current study was also investigated by Goopy et al. (2015); however, the previously predicted CH_4_ estimates were much closer to the actual CH_4_ value (±0.1 g/h), compared with the current study that tended to overestimate CH_4_ production (±0.03 l/h). While Goopy et al. (2015) prediction model was relatively consistent with the model used in the present study, the model in the current study did include extra variables on environmental conditions. Goopy et al. (2015) also used a larger dataset (706) compared with the present study (48).

Benaouda et al. (2020) tested various different prediction models for CH_4_ emissions in cattle and showed that including liveweight alone to predict CH_4_ resulted in a lower correlation between actual and predicted CH_4_ production (0.63) compared with the present study (0.71). Models used by Benaouda et al. (2020) found that feed intake was the key driver of variation in CH_4_ production with it accounting for 78% of variation and thus accounts for the large between-animal variance in CH_4_ production (Robinson et al., 2011). Including dry matter intake (**DMI**) or gross and digestible energy intake can increase the *r*^2^ for the CH_4_ prediction model to 0.77 (Yan et al., 2006) and 0.84 (Yan et al., 2000), respectively. DMI was not measured during the present study so DMI could not be included in the current prediction equation. Feed quality can also impact the number of methanogens available in the rumen, thus altering the pattern of fermentation (Kumar et al., 2009). The current study’s prediction equation overestimated gaseous production for all three gases; therefore, to increase the accuracy of future prediction equations, one would need to include information on diet quality and on DMI and base it off a larger dataset to increase the *r*^2^ value achieved.

## Conclusion

Gaseous measurements are moderately repeatable in the PAC over a 17 consecutive day period. For PAC to be used on larger-scale studies, the ranking of animals must remain consistent across time. In the current study, the ranking of animals remained consistent for 7 d. Within research trials where a second PAC measurement is required, the second measurement should be taken 7 d apart from the first measurement. A high level of precision was achieved when gaseous measurements from PAC were taken over three consecutive days. To develop an accurate and robust prediction equation for CH_4_ in sheep, a larger dataset is required along with additional variables such as DMI and feed quality.

## Abbreviations

ADF: acid detergent fiber
CP: crude protein
DM: dry matter
DMI: dry matter intake
GHG: greenhouse gas
NDF: neutral detergent fiber
PAC: portable accumulation chamber
RMSE: root mean square error
